# NMR-based metabolomic profiling can differentiate follicular lymphoma from benign lymph node tissues and may be predictive of outcome

**DOI:** 10.1038/s41598-022-12445-5

**Published:** 2022-05-18

**Authors:** Mohammad Mehdi Banoei, Etienne Mahé, Adnan Mansoor, Douglas Stewart, Brent W. Winston, Hamid R. Habibi, Meer-Taher Shabani-Rad

**Affiliations:** 1 Department of Critical Care Medicine, Cumming School of Medicine, University of Calgary, Calgary, AB Canada; 2 Department of Pathology and Laboratory Medicine, Foothills Medical Centre, Cumming School of Medicine, University of Calgary, McCaig Tower, Room MT7523, 1403 29 St NW, Calgary, AB T2N 2T9 Canada; 3 Departments of Oncology and Medicine, University of Calgary and Tom Baker Cancer Centre, Calgary, AB Canada; 4 Departments of Critical Care Medicine, Medicine and Biochemistry and Molecular Biology, University of Calgary, Calgary, AB Canada; 5 Department of Biological Sciences, University of Calgary, Calgary, AB Canada

**Keywords:** Laboratory techniques and procedures, Cancer metabolism, Prognostic markers, Lymphoma

## Abstract

Follicular lymphoma (FL) is a cancer of B-cells, representing the second most common type of non-Hodgkin lymphoma and typically diagnosed at advanced stage in older adults. In contrast to the wide range of available molecular genetic data, limited data relating the metabolomic features of follicular lymphoma are known. Metabolomics is a promising analytical approach employing metabolites (molecules < 1 kDa in size) as potential biomarkers in cancer research. In this pilot study, we performed proton nuclear magnetic resonance spectroscopy (^1^H-NMR) on 29 cases of FL and 11 control patient specimens. The resulting spectra were assessed by both unsupervised and supervised statistical methods. We report significantly discriminant metabolomic models of common metabolites distinguishing FL from control tissues. Within our FL case series, we also report discriminant metabolomic signatures predictive of progression-free survival.

## Introduction

Follicular lymphoma (FL) is one of the most frequently diagnosed lymphomas, representing nearly one-fifth of all lymphoma diagnoses^1^. FL is typically a diagnosis of older adults, with the median age of diagnosis in the seventh decade^1^. Data suggest that FL is slightly more prevalent in women and, based on US data, is far more frequently diagnosed in Caucasians than in African Americans^1^.

FL is a neoplasm of germinal center derived B-cells, recapitulating the architectural features of lymphoid follicles as an exuberant nodular proliferation^2^. In contrast to normal follicular B-cells, however, FL cells usually demonstrate the t(14;18) translocation^2^. FL often presents with extensive disease and may transform to more biologically aggressive lesions such as diffuse large B-cell lymphoma^1,2^.

There has been much effort in recent decades to better describe and understand the biology of FL; this research has focused mainly on exploring the molecular genetic profiles of FL^3–5^. Previous work has demonstrated that unique molecular genetic signatures can be employed to distinguish FL from other lymphomas, especially diffuse large B-cell lymphoma^4,5^. Other research has demonstrated a link between molecular genetic signatures and survival in FL^6^. Work has also evaluated the specific proliferative and immunologic milieu that may contribute to lymphomagenesis and aggressiveness in FL^7,8^. To our knowledge, however, work has not yet been undertaken to systematically investigate the metabolomic profile of FL.

Metabolomic profiling is the identification and quantification of sets of metabolites present in a cell or tissue^9^. High throughput technologies such as proton nuclear magnetic resonance spectroscopy (1H-NMR), gas/liquid chromatography mass spectrometry enable us to quantify hundreds of metabolites with a high sensitivity and specificity approach^10^. Metabolomic analyses, as applied to cancer research, can provide a snapshot of current metabolic activity—or derangement, as might occur in the context of cancer—as represented by metabolite signatures^11^. Metabolomics can be used to identify pathophysiological states that might be characterized by altered signatures and might further inform the effect of a pharmacologic intervention or other extrinsic factor^12^. The potential for biomarker discovery using such techniques has also been recently highlighted and metabolomic profiling has been performed in hematolymphoid contexts, highlighting some potentially useful biomarkers^9,13,14^. Metabolomic features with potential drug targets have been recently identified for Diffuse Large B-cell Lymphoma^15^. We aimed to use ^1^H-NMR-based techniques to differentiate patients with FL from non-FL nodal tissues based on differences in metabolomic signatures.

## Results

We were able to acquire 35 cryopreserved lymph node specimens involved with FL from the Alberta Cancer Research biorepository, collected over the course of approximately 10 years. Of these, sufficient isolates were available for analysis in only 29 cases. A control set of 11 reactive lymph node specimens, also obtained from the Alberta Cancer Research biorepository, were compared with the FL cohort. Table 1 details the FL clinical data, with control demographic data detailed in Table 2. The control and case series did not differ significantly by age (Kolmogorov–Smirnov D = 0.45; p = 0.08) or sex (Pearson chi-square = 1.58; p = 0.21).

**Table 1:** FL cases (n = 29): demographic, clinical, pathology and biomarker data.

Age at biopsy	Mean	59 years
	Median	57 years
	Range	31–83 years
Sex	Female	12 (41%)
	Male	17 (59%)
Grade	1–2	19 (66%)
	3	2 (7%)
	Data not available	8 (27%)
Clinical stage	I/II	9 (31%)
	III/IV	20 (69%)
Ki-67 proliferation index	< 30%	13 (45%)
	≥ 30%	4 (14%)
	Data not available	12 (41%)
FLIPI	Low-to-Intermediate Risk (FLIPI ≤ 2)	13 (44%)
	High-risk (FLIPI ≥ 3)	8 (28%)
	Data not available	8 (28%)
Lymph node bulk	All nodes ≦ 6 cm	12 (41%)
	Any node > 6 cm	8 (28%)
	Data not available	9 (31%)

**Table 2:** Controls: demographic data.

Age at biopsy	Mean	45 years
	Median	51 years
	Range	24–60 years
Sex	Female	7 (64%)
	Male	4 (36%)
History of cancer	Yes	5 (45%)
	No	6 (55%)

### Metabolic profiling to differentiate follicular lymphoma from reactive lymph node controls

Seventy-five metabolites were identified from ^1^H-NMR spectroscopy data by the ChenomX ^1^H-NMR software (see Supplementary Fig. S1 for example FL ^1^H-NMR spectrum). Identified metabolites were mostly comprised of amino acids, organic acids, sugars, and volatile organic compounds.

**Figure 1:**
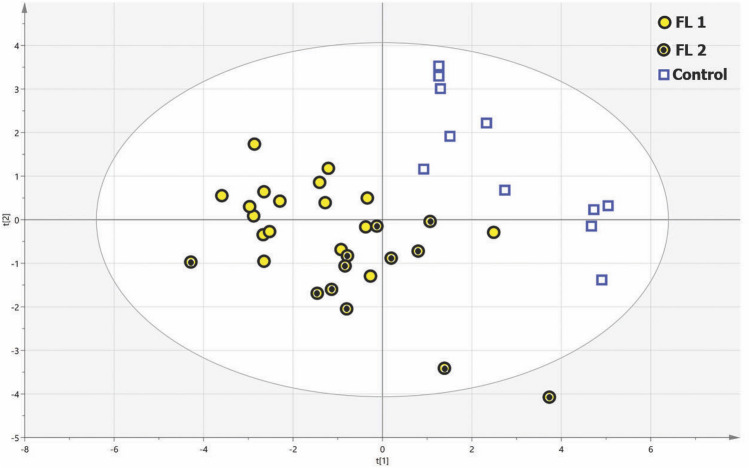
Partial least squares discriminant analysis highlights a very predictive (R^2^Y = 0.777, Q^2^Y = 0.639) and significant (p = 2.5e−07) separation between FL cases (including subsets FL1 and FL2) and controls using 28 metabolites (with a variable importance in projection > 1).

Good grouping of FL cases relative to controls was seen by PCA analysis (Supplementary Fig. S2) and there were no outlier specimens detected. The cumulative R^2^X of a 5 component PCA model was 0.49, with high variability among specimens. Similarly, OPLS-DA analysis discriminated FL cases from controls. By OPLS-DA modeling (p = 0.0004), the contribution of 24 metabolites separated the FL and control groups well (Q^2^Y = 0.431). PLS-DA results suggested the presence of unknown cofounder factor(s) influencing the OPLS-DA model, as PLS-DA provided more predictable (Q^2^Y = 0.639) and more significant (p = 2.5e−07) modeling using 28 metabolites. PLS-DA analysis also showed higher sensitivity and specificity compared to the OPLS-DA approach (Supplementary Table S1). The PLS-DA and OPLS-DA models are represented graphically in Fig. 1 and Supplementary Fig. S3, respectively. PLS-regression analyses demonstrated a strong correlation (R^2^ > 0.90) between the most discriminant metabolites and the separation of cases from controls by both OPLS-DA and PLS-DA analyses (Supplementary Fig. S4).

Coefficient plots suggested increased alanine, inosine, O-phosphocholine, threonine, nicotinamide adenine dinucleotide phosphate (NADPH), acetoacetate, fumarate, glucose, 2-hydroxybutyrate, taurine, lysine, 2-deoxygultarate, O-phosphoethanolamine, acetone, sn-glycero-3-phosphocholine, 4-hydroxybutyrate, adenosine diphosphate (ADP) and guanosine triphosphate (GTP) in FL cases when compared to controls. Moreover, dimethyl sulfone, uracil, formate, acetate, creatine, aspartate, creatinine ascorbate compounds were seen to be decreased in FL cases compared to the controls (Supplementary Fig. S5). An S-plot analysis demonstrated that inosine, taurine, O-phosphocholine, 2-deoxyglutarate, sn-glycero-3-phosphocholine, glutamate, 4-hydroxybutyrate, ADP and alanine could be more putative biomarkers with higher reliability and higher magnitude to separate FL from controls (Supplementary Fig. S6).

**Figure 2:**
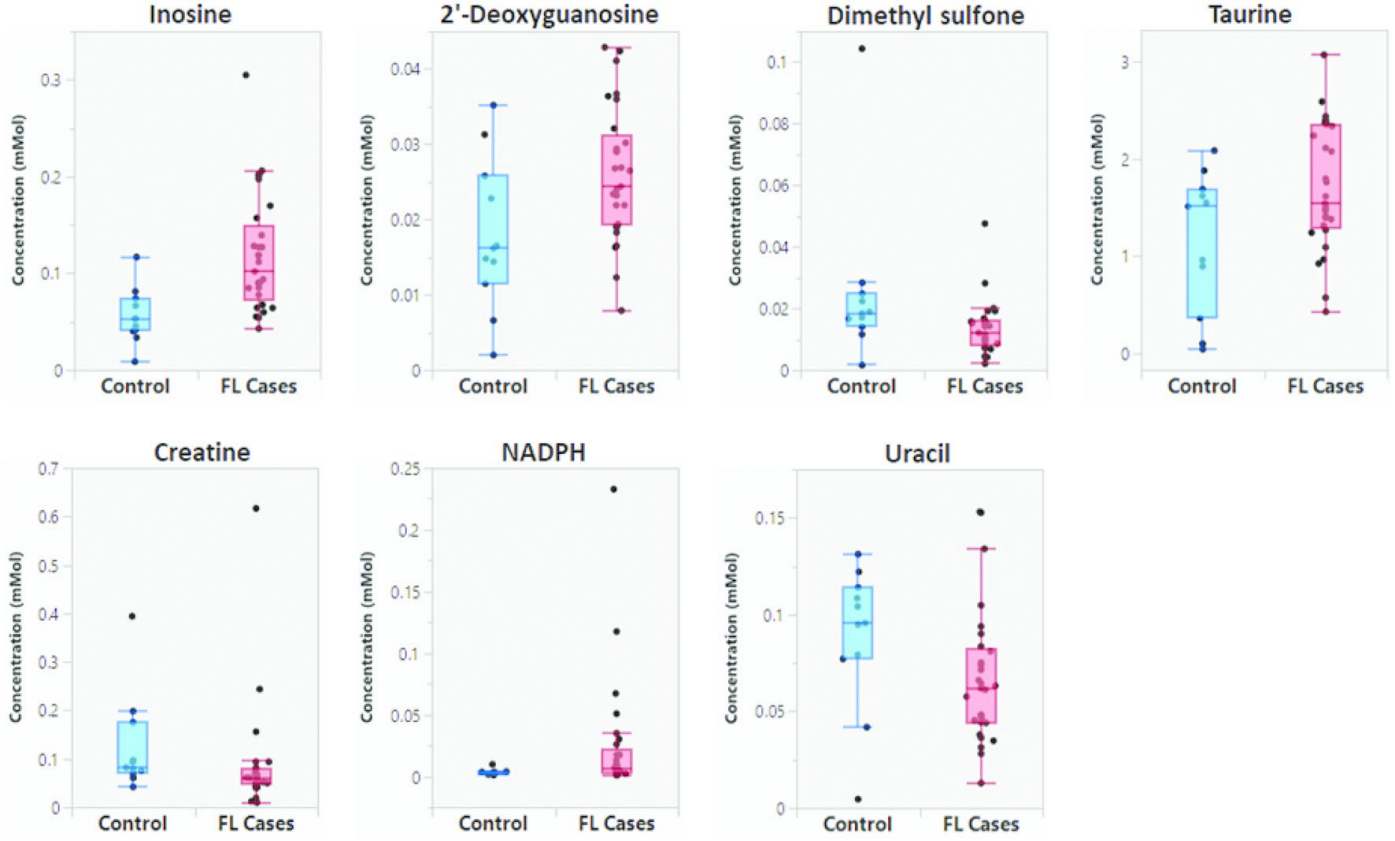
Dot and whisker plot showing the 7 metabolites highlighted by unpaired t-test analysis as being significantly different in follicular lymphoma cases relative to controls.

Using a univariate approach, unpaired t-test analysis identified 7 metabolites significantly enriched in FL cases (p < 0.05; Fig. 2) with one metabolite (inosine) also demonstrating a significant FDR (q < 0.05; Supplementary Table S2). The validity of R^2^ and Q^2^ values from the discriminant models were confirmed by permutation testing with 200 repetitions (Supplementary Fig. S7). This strongly suggests there is no overfitting of the data.

### Follicular lymphoma metabolomics in relation to clinical and outcome data

**Figure 3:**
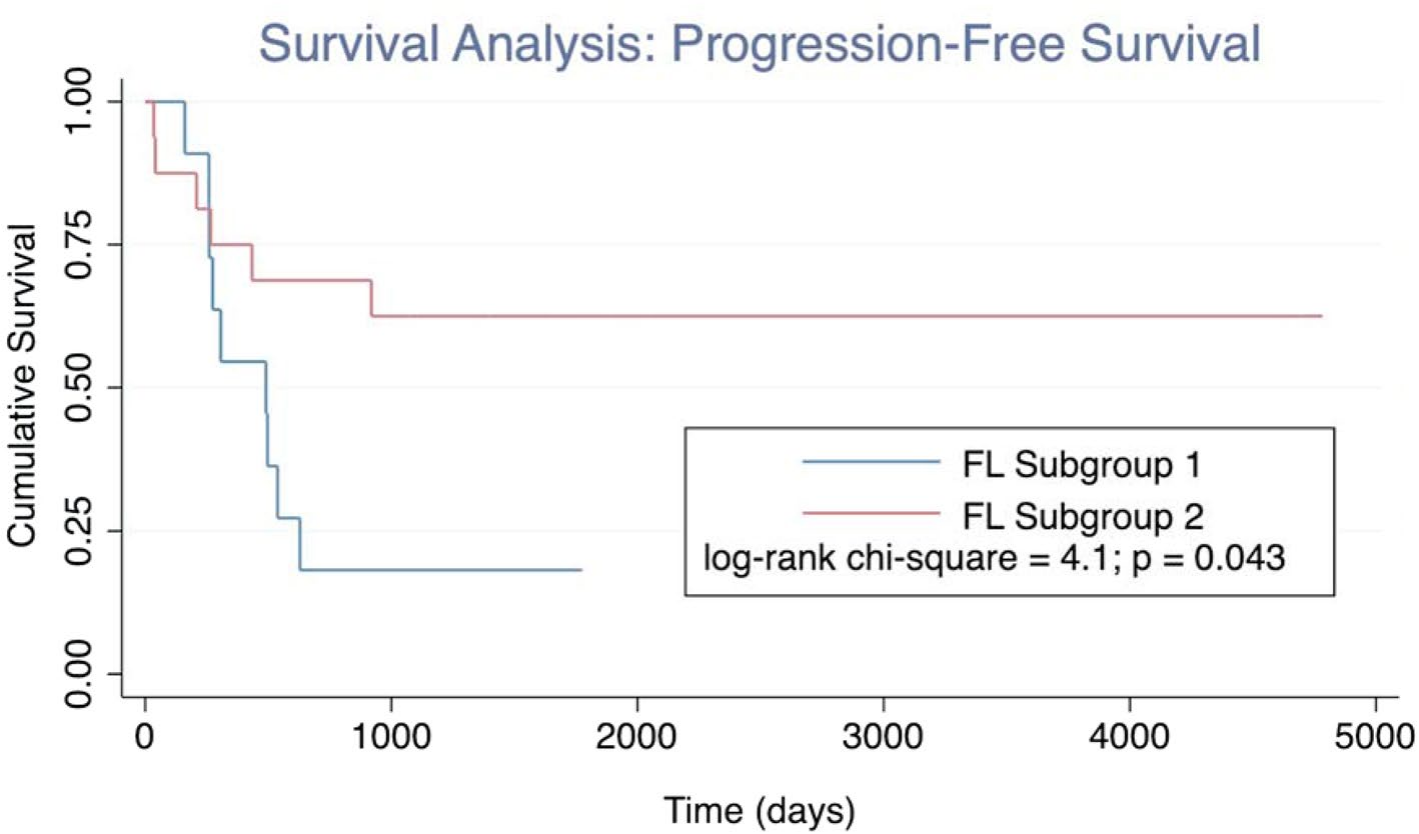
Kaplan–Meier analysis demonstrating significantly different progression-free survival by metabolomic subgroup.

In addition to distinct metabolomic differences seen when comparing FL cases to controls, we also identified two apparent FL subgroups (identified as FL1 and FL2 in Fig. 1) from among the cases. By the Kaplan–Meier method, we observed a significant difference in progression-free survival between the FL subgroups (log-rank chi-square = 4.1, p = 0.043; see Fig. 3). We also explored the potential impact of other clinical and pathology variables using widely accepted cut-offs to facilitate Kaplan–Meier analyses (see Supplementary Fig. S8A–G); none of these variables demonstrated significant prediction of progression-free survival in our case cohort, however. Notwithstanding our small sample size, only our metabolomic classifier demonstrated significance in multi-variate analyses (by the cox-proportional hazards method).

**Figure 4:**
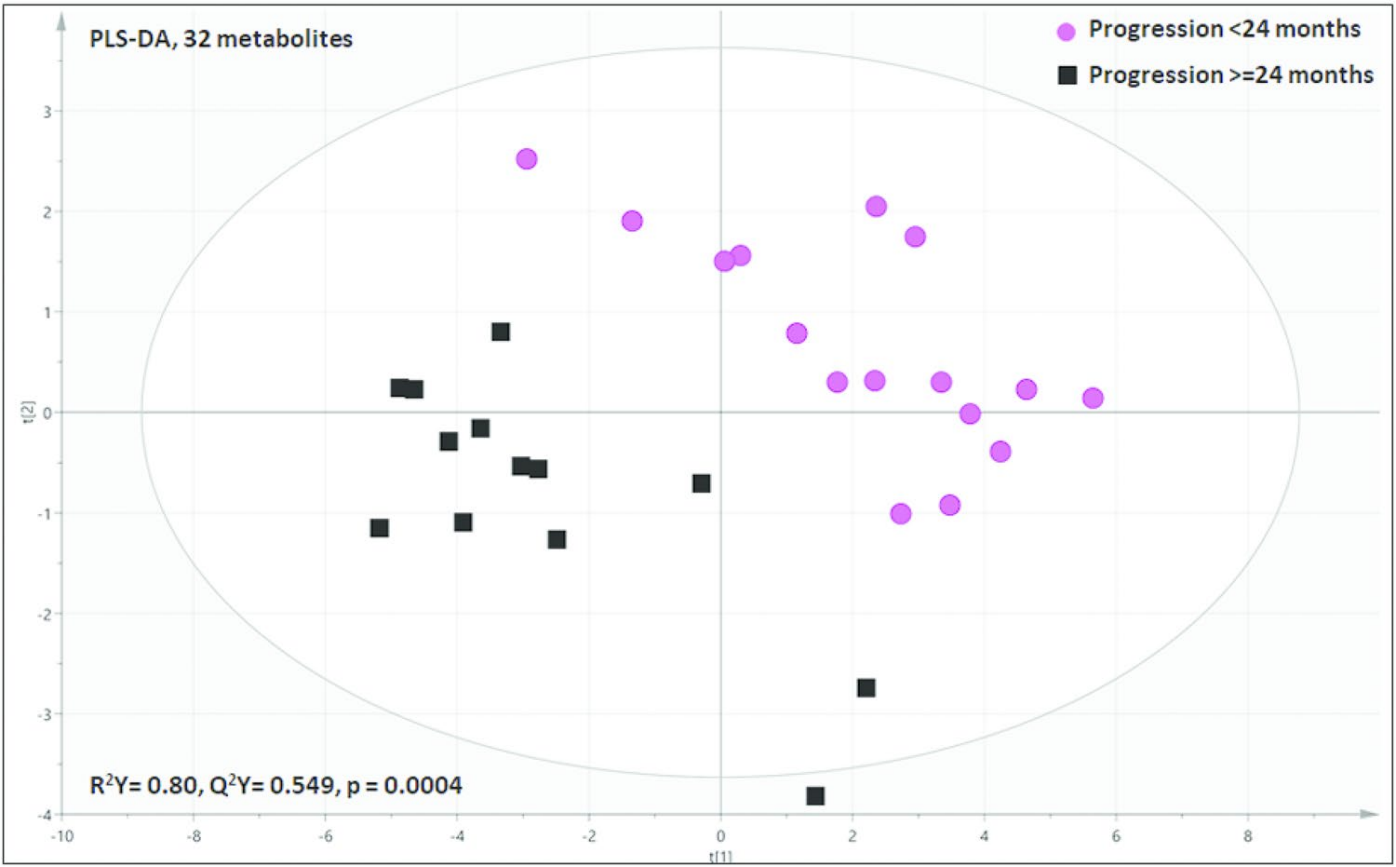
Partial least squares discriminant analysis highlighting good separation of FL cases with early progressive disease (< 24 months) and those who experienced later progressive disease (≥ 24 months) using 30 metabolites with the highest effect magnitude (Q2Y = 0.549; p = 0.0004).

To further explore the possible metabolites that might account for differential time-to-progression, we separated the FL cases into groups demonstrating progression at less than/greater than 24 months. In this manner, PLS-DA analysis noted a highly predictive and significant (Q^2^ = 0.549, and p = 0.0004) metabolomic model (Fig. 4). The FL cohort with early progression demonstrated increased mannose, glutamine, 2-oxoisocaporate, lysine, methanol, fumarate, alanine, beta-alanine, valine ornithine, leucine, tyrosine, ADP, isoleucine and AMP (Supplementary Fig. S9).

### Pathway analysis

The metabolomic profile of FL suggests upregulation of energy-conversion pathways, as evidenced by enrichment in FL cases for energy conversion pathway metabolites and intermediates. Increased levels of glucose, UDP-glucose, alanine, glutamate, ADP, GTP and NADPH suggest higher energy metabolism in FL cases compared to controls. Correspondingly, lower levels of uracil in FL cases suggest a relatively higher rate of DNA synthesis and cell growth relative to controls. Note is made of increased Inosine in the FL group vs the controls. This is indeed reflected in the MetaboAnalyst pathway analysis, demonstrating significantly upregulated aminoacyl-tRNA biosynthesis pathways and downregulated pyruvate metabolism pathways. Our data also suggested decreased pyruvate and increased lactate metabolites in FL cases compared to controls.

Increased branched chain amino acids and decreased antioxidant metabolites were associated with FL disease severity and early mortality. Multivariate analyses using clinical classification suggested increased valine, leucine and isoleucine in apparently more aggressive forms of FL. These branched chain amino acids were enriched in cases of FL demonstrating early progression of disease (progression at less than 24 months). We also observed a relative decrease in concentration of glutathione and hypoxanthine, which are known antioxidant and anticancer compounds^16^, in the more aggressive FL cases. Energy metabolism intermediates, such as sAMP and ADP and UDP-glucose, were also increased in the more aggressive FL cases.

## Methods

This study was reviewed and approved by the Health Research Ethics Board of Alberta (HREBA.CC16-0641); all experiments were performed in accordance with the relevant regulations of the Health Research Ethics Board of Alberta. Banked tissues were used as part of a materials transfer agreement undertaken between the principal investigator and the Alberta Cancer Research Biobank. Tissue banking procedures, and collection of clinical data, were performed with full and informed patient consent, and with oversight of our regional Institutional Review Board.

### Specimen selection and data collection

High-quality cryopreserved lymph node specimens from patients with Follicular Lymphoma, as well as cryopreserved lymph node specimens obtained from control (non-follicular lymphoma) patients, were obtained from our regional biobank. Where available, clinical data were also collected, including age at diagnosis, sex, stage, FL international prognostic index (FLIPI), primary site lymph node bulk (in cm), FL grade and Ki67 proliferation index. Fulsome overall survival data were sparse, owing in part to patient loss-to-follow-up; as such, our clinical outcome assessment was limited to progression-free survival.

### Specimen preparation and extraction of metabolites

We applied an adjusted methanol/chloroform method to extract metabolites from tissue specimens for ^1^H-NMR analysis^17^. One hundred mg of each cryopreserved specimen was transferred to 1.5 ml Eppendorf tubes followed by immediate addition of liquid nitrogen. Pre-cooled 200 µl methanol and 85 µl water were added to specimens in the tubes after evaporation of liquid nitrogen. Specimens were homogenized for 1 min at 3000 rpm using a homogenizer and resuspended in 200 µl chloroform and 200 µl water at 4 °C and vortexed for 15 min. Specimens were then centrifuged at 10,000 rpm for 15 min at 4 °C. The resulting aqueous and non-aqueous phases were analyzed separately.

Specimen volumes were adjusted to 400 µl with 80 µl of NMR buffer (0.5 M NaH_2_PO_4_ buffer solution at pH 7.0) containing 2.5 mM 2,2-dimethylsilapentane-5-sulfonate (DSS, final concentration 0.5 mM) as an internal reference compound, 10 µl sodium azide (1 M NaN_3_) to prevent bacterial growth, and deuterated water. The pH of the specimens was adjusted to 7.0 ± 0.04 and NMR assessment was performed at 4 °C.

### Metabolite quantification and profiling

^1^H-NMR data for all the specimens were generated in a blinded manner using a 600 MHz Bruker Ultrashield Plus NMR spectrometer (Bruker BioSPin Ltd., Canada). The one dimensional spectra were acquired using the Bruker 1D proton spectroscopy pre-saturation pulse sequence (noesy 1D) using an optimal water suppression program and a mixing time of 100 ms^18,19^. Initial specimens from each batch were sized to ensure the half-height line width of approximately 0.7–0.8 Hz for the DSS peak calibrated to 0.0 ppm. The NMR spectra were obtained with 1024 scans, zero filled and Fourier transformed to 128 k points. Obtained NMR spectra were corrected using line broadening, phasing, baseline correction and referencing to the DSS peak at 0.0 ppm using the Topspin software program (Bruker BioSpin Ltd., Canada). The ChenomX NMR Suite 7.1 software (ChenomX Inc., Edmonton, Alberta, Canada) was used to assign the metabolites peaks through processing and profiling modules, as has been described previously^18^. ^1^H-NMR spectra were manually phased followed by baseline correction. Water peak region was deleted from the spectra before metabolites alignment. The profiler module of ChenomX NMR Suite 7.1 was used to profile ^1^H-NMR spectra for metabolite identification and quantification using a non-targeted approach^18^. Metabolite identification was accomplished using the Human Metabolome Database (HMDB, version 2.5) linked to ChenomX, by matching the chemical shift patterns of metabolites in the query specimens with those in the library database, as described previously^20^. We used DSS as an internal standard for metabolite quantification (concentrations derived in mM)^21^.

### Data analysis

We performed multivariate and univariate data analyses to identify metabolite patterns. In the multivariate approach, ^1^H-NMR data were log transformed, mean-centered or scaled to univariance and then analyzed using the SIMCA-P + software (version 15.0.2, Umetrics AB, Umeå, Sweden). Unsupervised principal component analysis (PCA) was used to identify trends, for grouping and identification of outliers. Supervised partial least square discriminant analysis (PLS-DA) and orthogonal partial least discriminant analysis (OPLS-DA) were used to classify metabolites and for separation of distinct specimens. OPLS-DA can also identify potential confounding factors that appear as orthogonal components in the resulting statistical model, whereas PLS-DA will highlight distinct metabolomic profiles without influence of orthogonal factors.

The parameters R^2^Y, Q^2^Y and p values were considered when evaluating discriminant statistical models. R^2^Y and Q^2^Y represent goodness of model fit and goodness of predictability, respectively, and vary between 0 and 1. Cut off values of Q^2^Y > 0.3, > 0.5 and > 0.7 were established to define acceptable, good and excellent prediction models, respectively^22–24^. We generated S-plots to identify those putative metabolites with high model reliability and effect magnitude. Coefficient plots were generated to illustrate how metabolites change between specimen subgroupings in a relative correlation scale.

We used MetaboAnalyst (version 4.0)^21^ and Cytoscape (version 3.6.0)^25^ for pathway analysis using the most discriminant metabolites; metabolites with higher predictability (Q^2^Y) values were selected based on the OPLS-DA model. Clinical and survival statistical analyses were performed in Stata (version 17.0). Model p values less than 0.05 were considered significant.

## Discussion

To our knowledge, this is the first application of metabolomics to follicular lymphoma using ^1^H-NMR spectroscopy. ^1^H-NMR spectroscopy provides a reproducible and quantitative approach to metabolomic investigation, if perhaps less sensitive relative to mass spectrometry techniques, capable of providing a metabolomic snapshot including small polar molecules at micromolar concentrations. Although best seen as a pilot study of ^1^H-NMR in FL, our results suggest that an FL-specific metabolomic signature exists and that certain FL metabolomic patterns may be prediction of more aggressive disease. We observed a FL metabolomic profile enriching for pathways relating to energy metabolism. Elevated concentrations of ADP, AMP, GTP, NADHP, glucose and UDP-glucose tend to characterize FL relative to normal control tissues. We also report that elevated concentrations of branched-chain amino acids and lowered antioxidant and anti-inflammatory molecule tend to associate with more aggressive forms of FL.

Previous studies have reported elevated AMP, UDP, CTP, ATP, UTP and GMP in cancer cells^26,27^. Investigations on purines and pyrimidines in human lung cancer^28^ and breast, endometrial and prostate cancers^29^ have shown extracellular nucleosides (adenosine) and nucleotides (ATP, ADP, UTP, UDP and sugar UDP) could be correlated to disease progression as premetastatic and growth factors through the activation of purinergic signaling. Our data also suggest an enrichment of inosine in FL relative to benign tissues. Increased inosine has been observed in other cancers, and inosine has also been observed to stimulate tumor cell proliferation^30,31^. These observations may relate to impaired purine metabolism and increased cell turn-over^30,31^. Glycogen metabolism is reported to play an important role in the cancer microenvironment and elevated UDP-glucose and pentose phosphate pathway (PPP) characterize the relatively higher energy metabolism required by cancer cells^32^.

Other studies have also highlighted the significance of branched-chain amino acids in cancer growth, contributing to many biochemical and cell signaling pathways in both human^33,34^ and animal models of cancer^35^. Elevated concentrations of branched-chain amino acids have been associated with increased colorectal cancer risk^36^. Branched-chain amino acid deprivation (particularly of leucine) may impart an increased tendency for apoptosis^34^. Leucine enrichment has been shown to stimulate pancreatic cancer growth, for example^37^. Indeed, it has been suggested that enhanced glucose and branched-chain amino acid metabolism might portend a higher risk of metastasis of breast cancer to the brain^38^. In another study, plasma and urine levels of branched-chain amino acids were found to be relatively lower in prostate cancer cases than healthy controls^39^. Higher concentrations of leucine in prostate cancer tissues were found to associate with a higher rate of energy metabolism in models of human prostate cancer and in mouse cell lines^40^. Our data suggest that elevated branched-chain amino are associated with higher risk of progression in FL.

Perhaps not unexpectedly, our data also suggest relative depletion of glutathione and other antioxidant metabolites in more aggressive forms of FL. Glutathione plays different critical roles in cells, functioning as an antioxidant, balancing the intracellular redox state, modulating immune responses and assisting in detoxification of exogenous biological compounds^41^. Lower concentrations of glutathione in cancer cells might occur as a result of consumption of cystine/cysteine, glycine or glutamate^42^, and cancer cells deprived of glutathione are known to be relatively more sensitive to radiation^42,43^.

Certain study limitations should be highlighted. Owing mainly to the challenges of obtaining high-quality cryopreserved tissues, our FL cohort is small. Nevertheless, using banked cryopreserved tissues afforded us access to follow-up data, and available clinical information suggested that our cohort was reasonably representative. Of perhaps greatest import, however, is the need for independent validation of our results.

## Supplementary Information

Supplementary Information.

## Data Availability

The datasets used and/or analyzed as part of this study are available from the corresponding author on reasonable request. Requests will need to be vetted by the corresponding author’s regional institutional review board prior to data release.

## References

[1] Swenson WT (2005). Improved survival of follicular lymphoma patients in the United States. J. Clin. Oncol..

[2] Weltgesundheitsorganisation. *WHO classification of tumours of haematopoietic and lymphoid tissues*. (International Agency for Research on Cancer, 2017).

[3] Bende RJ, Smit LA, van Noesel CJM (2007). Molecular pathways in follicular lymphoma. Leukemia.

[4] Wang W (2012). MicroRNA profiling of follicular lymphoma identifies microRNAs related to cell proliferation and tumor response. Haematologica.

[5] Wang J (2002). Clustering of the SOM easily reveals distinct gene expression patterns: results of a reanalysis of lymphoma study. BMC Bioinf..

[6] Gentles AJ (2009). A pluripotency signature predicts histologic transformation and influences survival in follicular lymphoma patients. Blood.

[7] Janikova A (2011). Gene expression profiling in follicular lymphoma and its implication for clinical practice. Leuk Lymphoma.

[8] Dave SS (2004). Prediction of survival in follicular lymphoma based on molecular features of tumor-infiltrating immune cells. N. Engl. J. Med..

[9] Johnson CH, Ivanisevic J, Siuzdak G (2016). Metabolomics: Beyond biomarkers and towards mechanisms. Nat. Rev. Mol. Cell Biol..

[10] Banoei MM (2014). Metabolomics in critical care medicine: A new approach to biomarker discovery. Clin. Inestig. Med..

[11] Griffin JL, Shockcor JP (2004). Metabolic profiles of cancer cells. Nat. Rev. Cancer.

[12] Nicholson JK, Holmes E, Elliott P (2008). The metabolome-wide association study: A new look at human disease risk factors. J. Proteome Res..

[13] Zhou Q-Y (2014). Metabolomics investigation of cutaneous T cell lymphoma based on UHPLC-QTOF/MS. Asian Pac. J. Cancer Prev..

[14] Yang F (2017). Serum metabolomics of burkitt lymphoma mouse models. PLoS ONE.

[15] Cerchietti L (2012). Serum metabolomics uncovers a new therapeutic target in diffuse large B cell lymphoma (DLBCL. Blood.

[16] Bansal A, Simon MC (2018). Glutathione metabolism in cancer progression and treatment resistance. J. Cell Biol..

[17] Beckonert O (2007). Metabolic profiling, metabolomic and metabonomic procedures for NMR spectroscopy of urine, plasma, serum and tissue extracts. Nat. Protoc..

[18] Weljie AM (2006). Targeted profiling: Quantitative analysis of 1H NMR metabolomics data. Anal. Chem..

[19] Nicholson JK (1995). 750 MHz 1H and 1H–13C NMR spectroscopy of human blood plasma. Anal. Chem..

[20] Wishart DS (2009). HMDB: A knowledgebase for the human metabolome. Nucleic Acids Res..

[21] Xia, J. & Wishart, D. S. *Using MetaboAnalyst 3.0 for Comprehensive Metabolomics Data Analysis*. (Curr Protoc Bioinformatics, 2016). 10.1002/cpbi.11 27603023

[22] *Multi- and megavariate data analysis. 1: Basic principles and applications*. (MKS Umetrics AB, 2013).

[23] *Plant metabolomics: methods and applications*. (Springer, 2015).

[24] Peng DX, Lai F (2012). Using partial least squares in operations management research: A practical guideline and summary of past research. J. Oper. Manag..

[25] Shannon P (2003). Cytoscape: A software environment for integrated models of biomolecular interaction networks. Genome Res..

[26] Liu X (2014). LC-based targeted metabolomics analysis of nucleotides and identification of biomarkers associated with chemotherapeutic drugs in cultured cell models. Anticancer Drugs.

[27] Zhang C (2013). Targeted metabolic analysis of nucleotides and identification of biomarkers associated with cancer in cultured cell models. Acta Pharmaceut. Sin. B.

[28] Schneider G (2015). Extracellular nucleotides as novel, underappreciated pro-metastatic factors that stimulate purinergic signaling in human lung cancer cells. Mol. Cancer.

[29] Di Virgilio F, Adinolfi E (2017). Extracellular purines, purinergic receptors and tumor growth. Oncogene.

[30] Yin J (2018). Potential mechanisms connecting purine metabolism and cancer therapy. Front. Immunol..

[31] Chen J (2017). Inosine released from dying or dead cells stimulates cell proliferation via adenosine receptors. Front. Immunol..

[32] Zois, C. E. & Harris, A. L. Glycogen metabolism has a key role in the cancer microenvironment and provides new targets for cancer therapy. *J. Mol. Med. (Berl* **94**, 137–54 (2016). 10.1007/s00109-015-1377-9 PMC4762924 26882899

[33] Ananieva EA, Wilkinson AC (2018). Branched-chain amino acid metabolism in cancer. Curr. Opin. Clin. Nutr. Metab. Care.

[34] Xiao F (2016). Leucine deprivation inhibits proliferation and induces apoptosis of human breast cancer cells via fatty acid synthase. Oncotarget.

[35] Baracos VE, Mackenzie ML (2006). Investigations of branched-chain amino acids and their metabolites in animal models of cancer. J. Nutr..

[36] Budhathoki S (2017). Association of plasma concentrations of branched-chain amino acids with risk of colorectal adenoma in a large Japanese population. Ann. Oncol..

[37] Liu KA (2014). Leucine supplementation differentially enhances pancreatic cancer growth in lean and overweight mice. Cancer Metab..

[38] Chen J (2015). Gain of glucose-independent growth upon metastasis of breast cancer cells to the brain. Can. Res..

[39] Derezinski P (2017). Amino acid profiles of serum and urine in search for prostate cancer biomarkers: A pilot study. Int. J. Med. Sci..

[40] Billingsley KL (2014). The feasibility of assessing branched-chain amino acid metabolism in cellular models of prostate cancer with hyperpolarized [1-13C]-ketoisocaproate. Magn. Reson. Imaging.

[41] Balendiran GK, Dabur R, Fraser D (2004). The role of glutathione in cancer. Cell Biochem. Funct..

[42] Ortega AL, Mena S, Estrela JM (2011). Glutathione in cancer cell death. Cancers.

[43] Traverso, N. Role of glutathione in cancer progression and chemoresistance. *Oxid. Med. Cell Longev.* 972913 (2013). 10.1155/2013/972913 PMC3673338 23766865

