# Supplementary material for: NMR-based metabolomic profiling can differentiate follicular lymphoma from benign lymph node tissues and may be predictive of outcome
Source: Sci Rep. 2022 May 18;12:8294. doi: 10.1038/s41598-022-12445-5 (PMC9117304; doi:10.1038/s41598-022-12445-5)

Supplementary Figure S1. Example ^1^H-NMR spectrum from an FL specimen, highlighting peak assignments


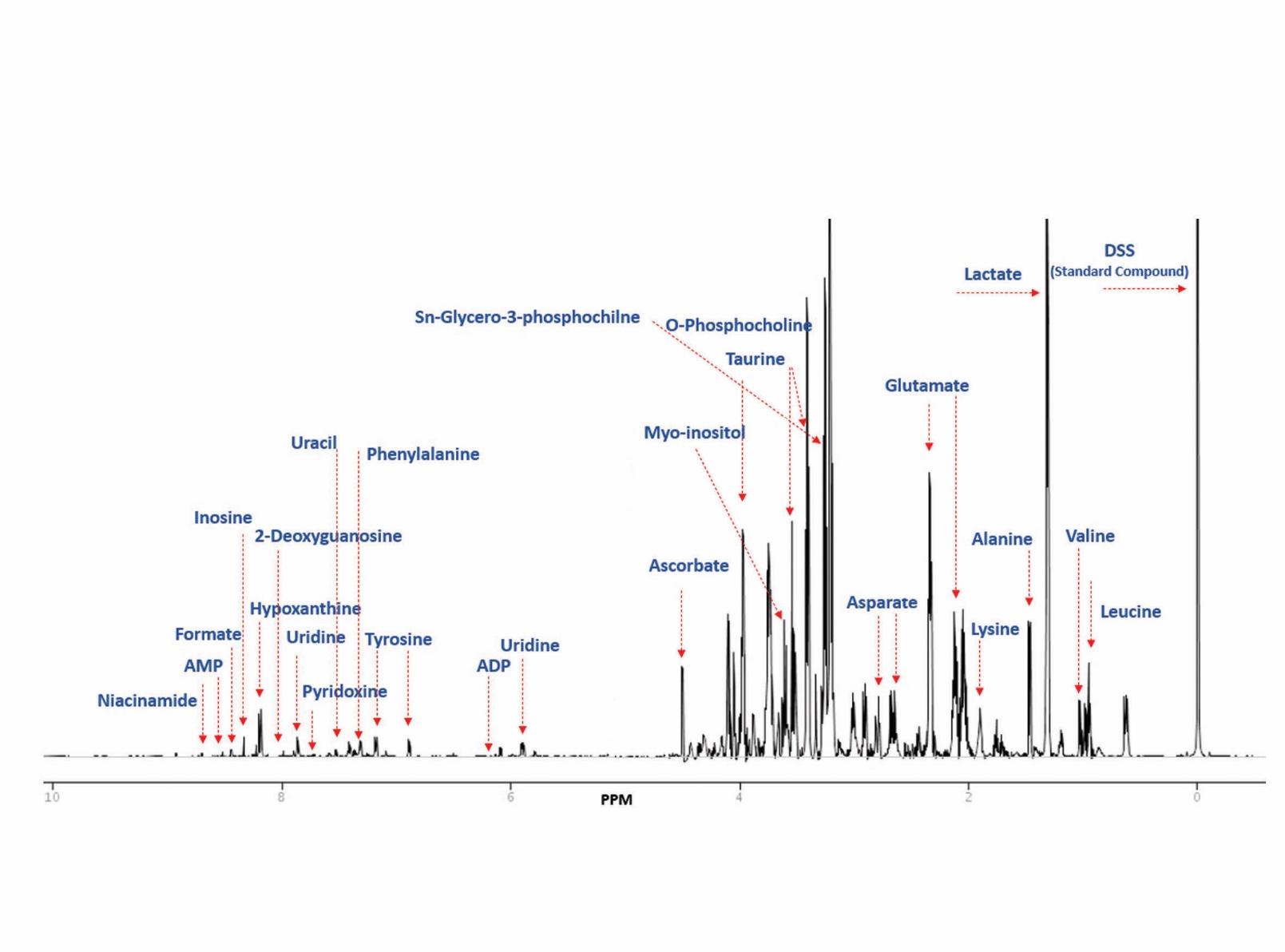


Supplementary Figure S2. PCA analysis highlights the grouping of FL cases relative to controls (RLNs) using ^1^H-NMR analysis on all 75 identifiable metabolites


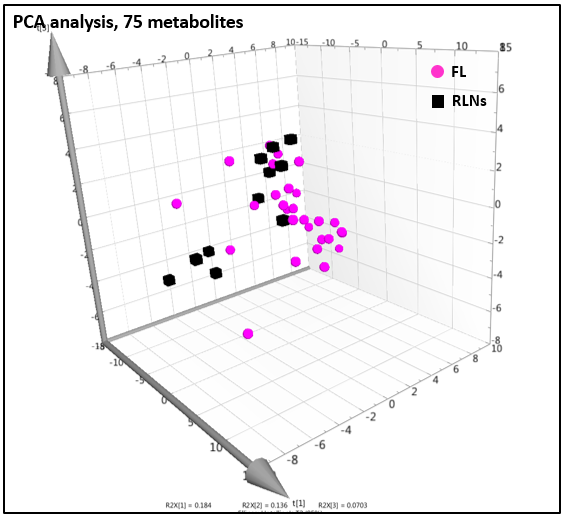


Supplementary Table S1. The characteristics of OPLS-DA and PLS-DA models to separate FL patients from controls; PLS-DA results appear to suggest a more predictive, significant, sensitive and specific statistical model when compared to the OPLS-DA model. OPLS-DA used 24 metabolites with variable importance in projection (VIP) > 1) to build the model. PLS-DA used 28 metabolites (VIP > 1) to build the model.

| Model | R^2^Y | Q^2^Y | *p* value | Sensitivity | Specificity | AUC ROC | R^2^ regression |
| --- | --- | --- | --- | --- | --- | --- | --- |
| OPLS-DA | 0.674 | 0.431 | 0.0004 | 81 | 93 | 0.98 | 0.90 |
| PLS-DA | 0.777 | 0.639 | 2.5e-07 | >99% | 96 | >0.99 | 0.90 |

Supplementary Figure S3. OPLS-DA analysis highlights a good predictive (Q^2^Y= 0.573) and significant (p value = 0.002) separation between FL cases and controls using 24 metabolites


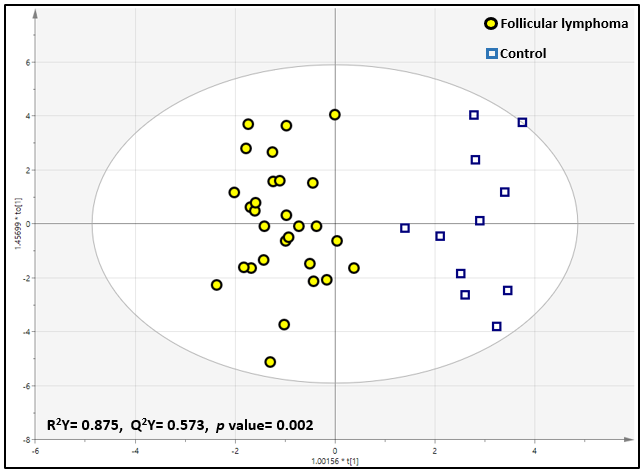


Supplementary Figure S4. PLS-regression analysis highlighting a very strong correlation (R^2^=0.90) between the metabolites with the highest effect magnitude and the separation of FL cases from controls.


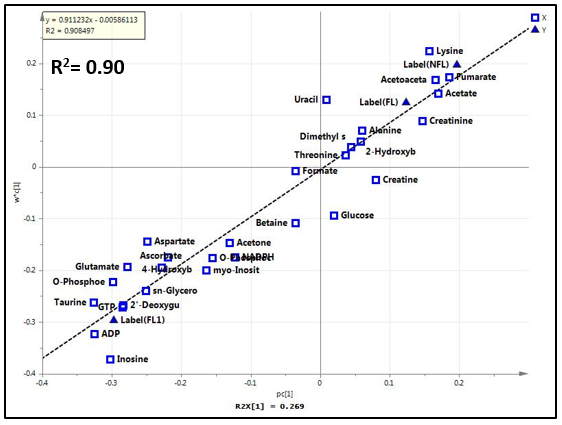


Supplementary Figure S5. Coefficient plot showing the relative correlation of metabolite changes in FL vs. controls. Only those metabolites with a VIP > 1 are shown.


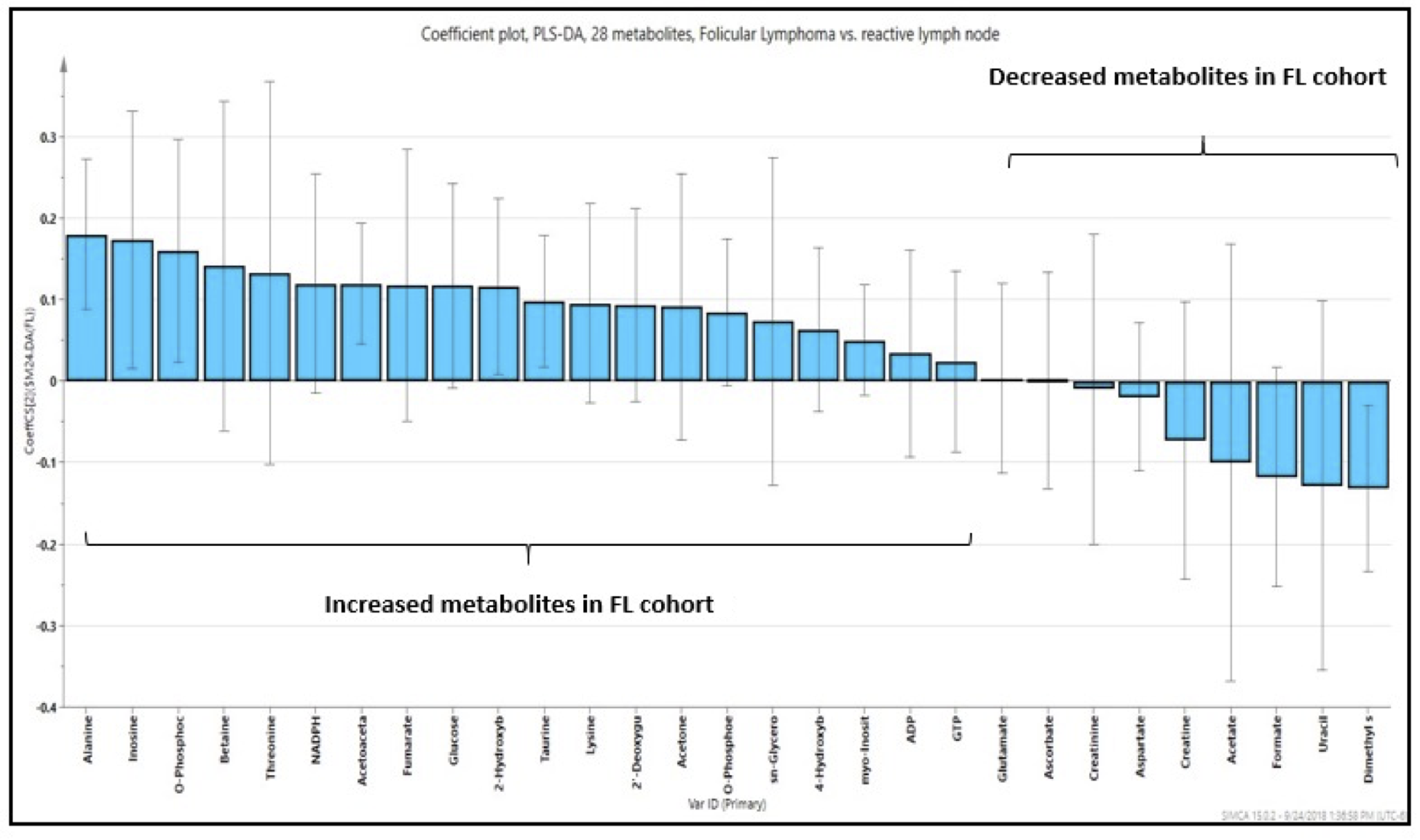


Supplementary Figure S6. S-Plot highlighting the metabolites with the highest effect magnitude in the separation of the FL cohort relative to controls.


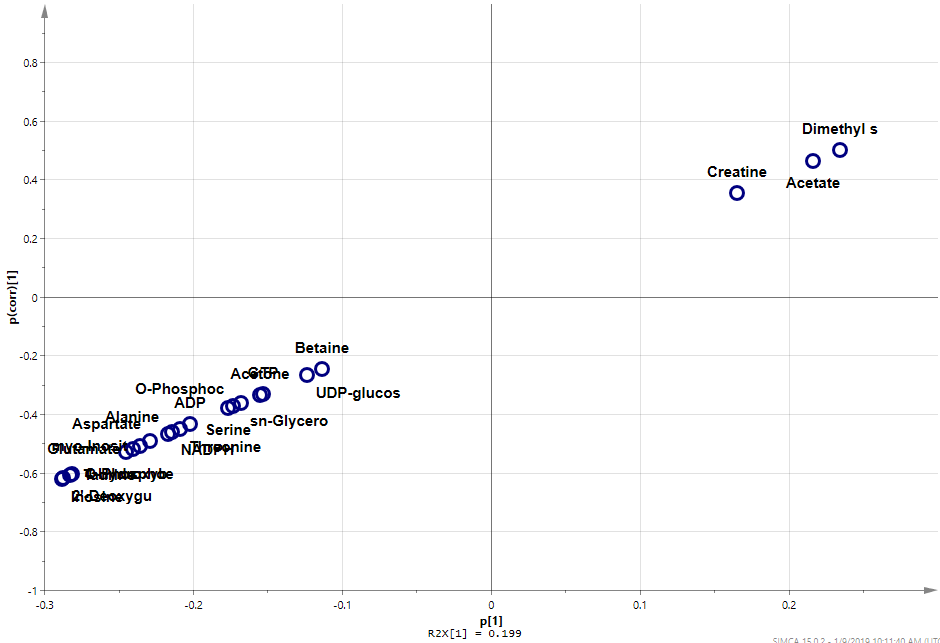


Supplementary Table S2. Unpaired t-test of metabolite concentrations in FL cases relative to controls.

| Name | Mean (SD) of Follicular Lymphoma | Mean (SD) of Control | p-value | q-value (FDR) | Fold Change |
| --- | --- | --- | --- | --- | --- |
| 2'-Deoxyguanosine | 0.026 (0.009) | 0.018 (0.010) | 0.0158 | 0.3553 | 1.46 |
| Taurine | 1.708 (0.641) | 1.153 (0.726) | 0.0235 | 0.3553 | 1.48 |
| Inosine | 0.118 (0.060) | 0.056 (0.028) | 0.0001 (W) | 0.0089 | 2.12 |
| Creatine | 0.085 (0.112) | 0.125 (0.101) | 0.0189 (W) | 0.3553 | -1.46 |
| Dimethyl sulfone | 0.013 (0.009) | 0.025 (0.027) | 0.0240 (W) | 0.3553 | -1.88 |
| NADPH | 0.024 (0.047) | 0.003 (0.002) | 0.0394 (W) | 0.4013 | 6.96 |
| Uracil | 0.067 (0.035) | 0.088 (0.037) | 0.0412 (W) | 0.4013 | -1.32 |

Supplementary Figure S7. Permutation test demonstrating the validity of R^2^ and Q^2^ derived from PLS-DA analysis of the separation of FL cases and controls. This strongly suggests there is no overfitting of the data.


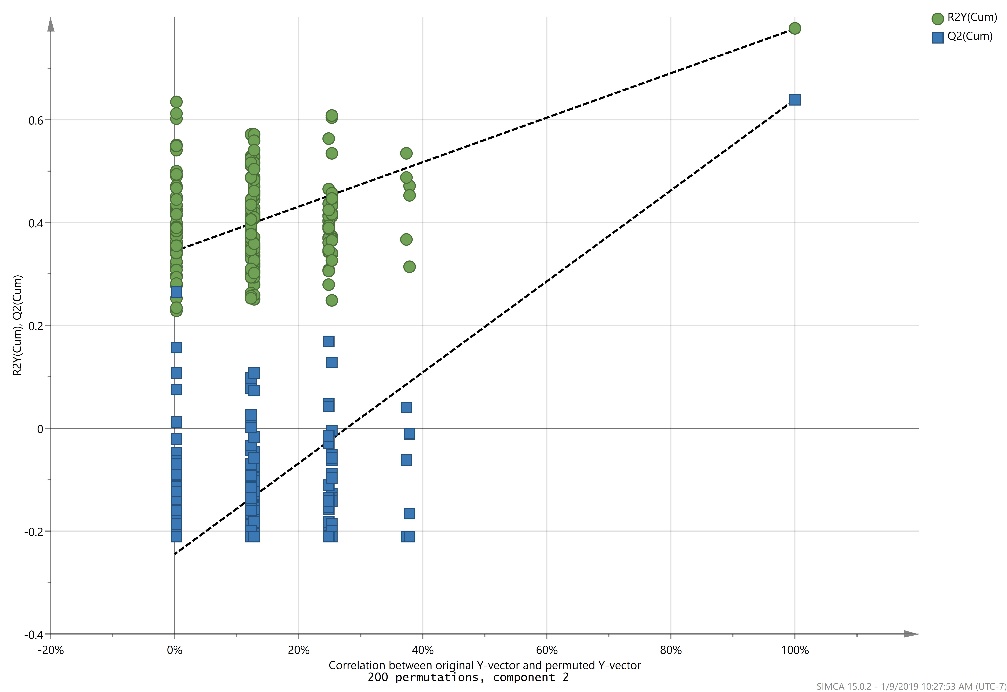


Supplementary Figure S8: Kaplan-Meier analyses: A: Age (by median); B: Sex; C: FL Grade; D: Ki-67 Proliferation Index (less than/greater than or equal to 30%); E: FL Stage (Low/High); F: Lymph Node Bulk (largest nodal region less than/greater than or equal to 6 cm); G: FLIPI ( ≤2 or ≥3)







B

A







D

C







F

E





G

Supplementary Figure S9. Coefficient plot highlighting the relative concentration of metabolite changes in FL patients with early progressive disease (less than 24 months) vs. late progressive disease (at or beyond 24 months). Only those metabolites with a VIP > 1 are shown.


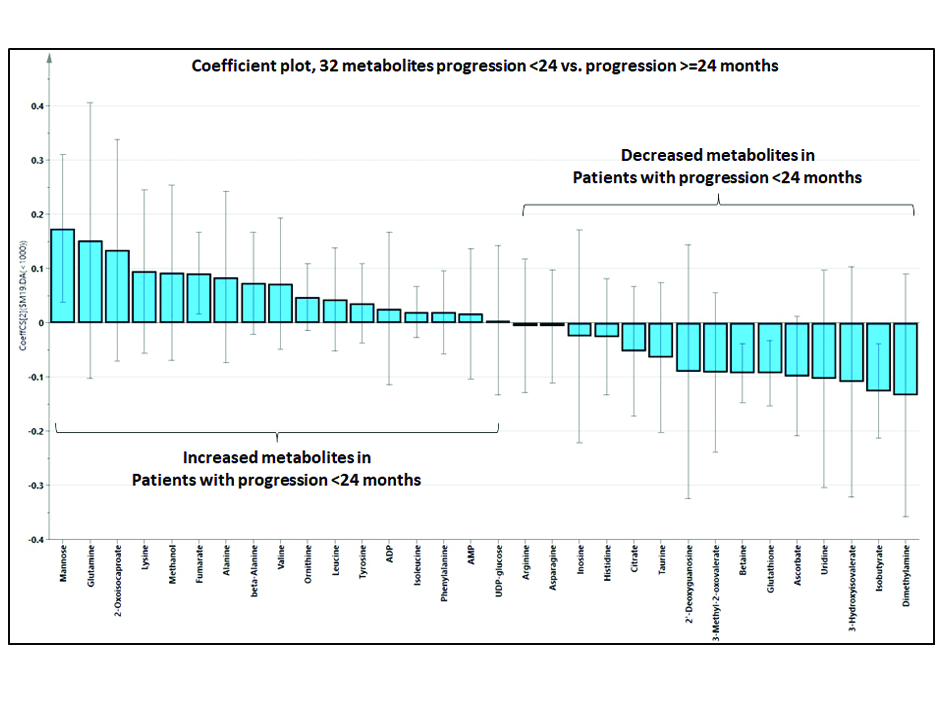

Supplement: Supplementary file 1 — Supplementary Information. [file 41598_2022_12445_MOESM1_ESM.docx]
